# Determinants of Hospital-based Physician Participation in Quality Improvement: A Survey of Hospitalists in British Columbia, Canada

**DOI:** 10.4103/JQSH.JQSH_17_19

**Published:** 2020-02-06

**Authors:** Vandad Yousefi, Alaleh Asghari-Roodsari, Sarah Evans, Cynthia Chan

**Affiliations:** 1 Department of Family Practice, University of British Columbia, Vancouver, British Columbia, Canada; 2 Hospitalist Program, Vancouver General Hospital, Vancouver, British Columbia, Canada; 3 VIP Doctor 247, Dubai, United Arab Emirates; 4 Royal Roads University, Victoria, British Columbia, Canada; 5 Sarah Evans Coaching and Consulting, Victoria, British Columbia, Canada; 6 Department of Family Practice, University of British Columbia, Vancouver, British Columbia, Canada; 7 Department of Family and Community Practice, Vancouver Community, Vancouver Coastal Health Research Institute, Vancouver, British Columbia, Canada

**Keywords:** Hospitalist medicine, physician engagement, quality improvement

## Abstract

**Objective:**

We aimed to understand the extent of hospitalist involvement in system improvement efforts across the province of British Columbia in Canada and provide insights into determinants of such participation.

**Materials and Methods:**

We designed a web-based survey and asked about individual, programmatic, and institutional characteristics that may facilitate or impair hospitalist involvement in quality improvement (QI) activities. The survey was sent to all individuals who participated in “hospitalist care” from January 2014 to February 2015, in the province of British Columbia, Canada. We conducted both quantitative and qualitative analysis of responses.

**Results:**

We received 57 complete responses to the survey of 322 invited individuals (17.7% response rate). Of these, 15 individuals (26.3%) indicated that they had participated in QI initiatives. Respondents highlighted high clinical workload and lack of time, lack of QI skills and training, lack of access to performance data, poor support from hospital/health authority administration, and lack of financial compensation as main barriers to QI involvement. These themes were also supported in logistic regression, where QI training and the number of weeks worked as a hospitalist showed significant predictive properties for involvement in QI initiatives.

**Conclusion:**

Our study attempts to understand the various individual or organizational attributes that could facilitate involvement by hospital-based generalist physicians in QI activities. Our findings show lack of formal QI training is an important barrier for hospitalist involvement in QI, and highlight the need for formal training, dedicated time, support from physician leadership, and financial incentive as important facilitators for participation in systemic improvement efforts.

## Introduction

Active participation by physicians in quality improvement (QI) projects (“physician engagement”) has been widely considered to be a necessary prerequisite to ensure a project's success, just as lack of buy-in from physicians can negatively affect the adoption of new care processes.[1–5]

Since the early 1990s, “hospitalists” have emerged as one of the fastest growing physician specialties in North America.[6–8] Hospitalists are generalist physicians “whose primary professional focus is the general medical care of hospitalized patients.”[9] Most hospitalists have training backgrounds that are rooted in the principles of family medicine. As they spend the majority of their clinical time in acute care facilities, hospitalists have a unique opportunity to engage in QI.[10] Anecdotal evidence suggests that across Canada, hospitalists are leading QI projects. For example, hospitalists in British Columbia (BC) implemented a QI collaborative across 11 hospital medicine programs that increased venous thromboembolism prophylaxis rates from 58% to 93%.[11] National surveys also suggest that 26.2% of respondents spent at least 5% of their time on QI initiatives.[12]

Although there is a robust literature examining the factors that impact “physician engagement” generally, less is known about potential differences that may exist in levels of engagement between different specialties.[4] Specifically, our understanding about the actual levels of engagement in QI initiatives by hospitalists is limited. We also know little about individual, programmatic, and institutional characteristics that may facilitate or impair such involvement, as well as the perceptions of hospitalist physicians about barriers and facilitator of participation in systemic change efforts. Given that the introduction of the hospitalist model has been a major departure from the traditional inpatient care model,[13,14] it is possible that physicians who choose to work as hospitalists have a different level of interest in engaging with their organizations compared to other specialties.

In the current research, we aimed to understand the extent of hospitalist involvement in system improvement efforts across the province of BC in Canada and to provide insights into determinants of such participation. We attempted to explore a number of questions about this engagement: are there specific personal characteristics (e.g., age, work experience, and training) that would facilitate an individual's participation in system improvement? Are there certain attributes about the hospitalist program (such as how many individuals work in the program, how long it has been in operation, and individual clinical workload) that allow for more QI involvement? Does the type of institution (e.g., large vs. small hospital and academic vs. community) in which these programs operate have an impact? We also attempted to understand how individual hospitalists perceived factors that facilitated or impaired their engagement with their health-care institutions.

## Materials and Methods

### Survey design

We designed a structured web-based survey of individuals who participated in “hospitalist care” in BC from January 2014 to February 2015. The questionnaire items were developed based on similar previously validated surveys used in prior studies[15,16] and a review of literature.[17–22] The survey contained questions about individual characteristics such as demographic information, work experience, as well as full-time and part-time work status. Individuals were also asked about their involvement in QI activities, the amount of time they dedicated to such projects, and other information (such as compensation schemes, the types of QI projects involved, and formal training in improvement methodologies). In addition, we asked questions about programmatic and organizational attributes. Respondents were asked to rank in the order of importance factors that have been previously suggested in the literature as barriers or facilitators of QI involvement, and additional freehand text boxes provided additional opportunity to collect comments. We also explored respondents' views on the effectiveness of common improvement strategies such as use of computer technologies or treatment protocols (we have previously presented this analysis elsewhere[23]). The detailed questionnaire can be found in Appendix A. (available online only as supplemental material)

We used the Enterprise Feedback Management system (Verint Systems, Melville, New York) to develop our survey. Individuals were asked for informed consent at the start of the web-based survey. The survey was anonymous and the responses could not be matched to specific individual participants. Incomplete responses were omitted from the final analysis. Follow-up reminder e-mails were sent at 2 and 4 weeks, and the survey was closed 6 weeks after the initial date.

### Participants

To develop a comprehensive list of “hospitalists” in the province, we contacted all hospitalist department or division heads in BC and invited members of their programs to participate in our study in accordance to Canadian anti-spam legislation.[24] The list generated through this voluntary mechanism was complemented by the contact list maintained by the Section of Hospital Medicine of Doctors of BC, the professional association representing hospitalists in the province. The section leadership endorsed our study and provided a research grant. All individuals who participated in providing hospitalist care in the province in the preceding year were eligible for the study. Exclusion criteria included individuals who worked in hospitals without formal hospitalist programs, individuals for whom we could not obtain valid e-mail addresses or other contact information, and those who declined to provide their contact information for inclusion in the study. This study was approved by the University of British Columbia Behavioural Research Ethics Board.

### Analysis

We used a combination of qualitative and quantitative methods to analyze survey responses. To understand perceptions of respondents (qualitative analysis), we incorporated a number of rank-order questions. We analyzed these questions by assigning a score to each response and subsequently calculated mean values and standard deviations. For example, we asked respondents to rank in the order of importance (1 = most important and 8 = least important), eight barriers to participation in QI activities previously described in the literature. Each of the eight options in the previous example was given a score for each of the responses received corresponding to the rank chosen by the respondent. The survey also included a number of open-ended questions. We analyzed the freehand text answers using constant comparative analysis to uncover any additional themes not captured in the suggested ranking responses. Two members of the research team independently coded the texts and reviewed the findings jointly to arrive at a final set of identified themes. We subsequently compared the themes that emerged from this analysis with the responses to the rank-order questions.

In our quantitative analysis, we used nonparametric techniques to analyze relationships between levels of hospitalist participation in QI and various individual, programmatic, and institutional attributes. Given the categorical nature of attributes, chi-square tests were used to establish the extent to which cell distributions in the cross-tabulations between categorical variables were not due to chance. In instances where the chi-square test was significant, additional nonparametric statistics were applied to assess the strength of the relationship between the categorical variables. The λ (proportional reduction in error statistic) measure of association was the primary metric used to assess relationships. The λ measure was used because of the combined use of nominal and ordinal levels of measurement for the variables used in the analysis. We used a less conservative measure of significance; the significance level was set at *p* < 0.10. This level was used to provide some flexibility in identifying potential candidates for inclusion in logistic regression given the small sample sizes and the relative imbalance in the distribution between groups within some of the core variables. In an effort to reduce these limitations, we also collapsed similar categories. For example, we combined individuals working part time or as locums into one category, contrasting with those working full time. Factors that showed a significant χ^2^ result (defined as *p* < 0.10), as well as others that could potentially affect involvement in QI activities (such as sex, years of experience working as a physician, and medical training background) were then used in logistic regression to explore interactions between variables and their impact on QI participation. Finally, we compared these results with the outcomes of our qualitative analysis to see if the findings from the regression analysis confirmed or supported the insights gained from the qualitative approach.

We used the SPSS software (version 20, IBM, New York, USA) to perform statistical analyses.

## Results

### General findings

A total of 322 individuals were invited to participate in the study. We received 57 complete responses to the survey (response rate 17.7%). Of these, 15 individuals indicated that they had participated in QI initiatives (26.3% of respondents). Table 1 summarizes the various characteristics of study participants. Table 2 outlines additional details about various aspects of QI involvement for individuals who participated in such activities.

**Table 1:** Characteristics of survey respondents

**Characteristics**	**Individuals involved in not involved system redesign**	**Individuals in system redesign**
	
	***N* (%)**	***N* (%)**
Number of respondents	15 (26.3)	42 (73.7)
Male	11 (73.3)	25 (59.5)
Age (years)		
Less than 30	0 (0.0)	1 (2.4)
30–40	3 (20.0)	13 (31.0)
41–50	4 (26.7)	11 (26.2)
51–60	5 (33.3)	11 (26.2)
61–70	2 (13.3)	6 (14.3)
Over 70	1 (6.7)	0 (0.0)
Years in clinical practice as physician		
Less than 5	0 (0.0)	8 (19.1)
6–15	5 (33.3)	11 (26.2)
16 or more	10 (66.7)	23 (54.5)
Years working as hospitalist		
<6	0 (0.0)	16 (38.1)
6–10	7 (46.7)	15 (35.7)
>10	8 (53.3)	11 (26.2)
Credentials		
CCFP[1]	9 (60.0)	21 (50.0)
CCFP+ additional training	2 (13.3)	7 (16.7)
Other	4 (26.7)	10 (23.8)
Work status		
Full time	15 (100.0)	31 (73.8)
Part time or locum	0 (0.05)	11 (26.2)
Weeks worked in hospitalist program annually		
<25	0 (0.0)	9 (15.8)
25–36	3 (20.0)	21 (36.8)
>36	12 (80.0)	27 (47.4)
Weeks worked in clinical care annually		
Less than 24	0 (0.0)	2 (4.8)
25–36	2 (13.3)	13 (31.0)
37–48	12 (80.0)	25 (59.5)
49 or more	1 (6.7)	2 (4.8)
Daily average number of patients seen by an individual		
13–16	6 (40.0)	11 (26.2)
17–20	3 (20.0)	22 (52.4)
20 or more	6 (40.0)	9 (21.4)
Formally trained in quality measurement or improvement		
Yes	6 (40.0)	5 (11.9)
No	9 (60.0)	37 (88.1)
Role in hospitalist program		
Program lead	3 (20.0)	5 (11.9)
Quality improvement lead	1 (6.7)	4 (9.5)
CME coordinator	1 (6.7)	2 (4.8)
Committee participant	6 (40.0)	17 (40.5)
Program scheduling	4 (26.7)	3 (7.1)
Other	6 (40.0)	19 (45.2)
Hospital type		
Rural hospital	0 (0.0)	0 (0.0)
Small community hospital	0 (0.0)	2 (4.8)
Medium community hospital	6 (40.0)	12 (28.6)
Large community hospital	4 (26.7)	15 (35.7)
Academic hospital	5 (33.3)	13 (31.0)
Average daily census of hospitalist program		
Less than 100 patients	0 (0.0)	5 (11.9)
101–120 patients	0 (0.0)	1 (2.4)
121–140 patients	3 (20.0)	6 (14.3)
141 or more patients	12 (80.0)	30 (71.4)
Number of FTE in the program		
Less than 10	1 (6.7)	6 (14.3)
11–15	1 (6.7)	7 (16.7)
15 or more	13 (86.7)	25 (59.5)
How many years since hospitalist program was implemented		
Less than 5	0 (0.0)	0 (0.0)
6–10	1 (6.7)	8 (19.0)
11–15	13 (86.7)	26 (61.9)
16 or more	1 (6.7)	2 (4.8)
Not known	0 (0.0)	6 (14.3)

CCFP = Certificate of the College of Family Physicians of Canada, CME = continuing medical education, FTE = full time equivalent

**Table 1:** Continued

**Characteristics**	**Individuals involved in not involved system redesign**	**Individuals in system redesign**
	
	***N* (%)**	***N* (%)**
Number of respondents	15 (26.3)	42 (73.7)
Male	11 (73.3)	25 (59.5)
Age (years)		
Less than 30	0 (0.0)	1 (2.4)
30–40	3 (20.0)	13 (31.0)
41–50	4 (26.7)	11 (26.2)
51–60	5 (33.3)	11 (26.2)
61–70	2 (13.3)	6 (14.3)
Over 70	1 (6.7)	0 (0.0)
Years in clinical practice as physician		
Less than 5	0 (0.0)	8 (19.1)
6–15	5 (33.3)	11 (26.2)
16 or more	10 (66.7)	23 (54.5)
Years working as hospitalist		
<6	0 (0.0)	16 (38.1)
6–10	7 (46.7)	15 (35.7)
>10	8 (53.3)	11 (26.2)
Credentials		
CCFP[1]	9 (60.0)	21 (50.0)
CCFP+ additional training	2 (13.3)	7 (16.7)
Other	4 (26.7)	10 (23.8)
Work status		
Full time	15 (100.0)	31 (73.8)
Part time or locum	0 (0.05)	11 (26.2)
Weeks worked in hospitalist program annually		
<25	0 (0.0)	9 (15.8)
25–36	3 (20.0)	21 (36.8)
>36	12 (80.0)	27 (47.4)
Weeks worked in clinical care annually		
Less than 24	0 (0.0)	2 (4.8)
25–36	2 (13.3)	13 (31.0)
37–48	12 (80.0)	25 (59.5)
49 or more	1 (6.7)	2 (4.8)
Daily average number of patients seen by an individual		
13–16	6 (40.0)	11 (26.2)
17–20	3 (20.0)	22 (52.4)
20 or more	6 (40.0)	9 (21.4)
Formally trained in quality measurement or improvement		
Yes	6 (40.0)	5 (11.9)
No	9 (60.0)	37 (88.1)
Role in hospitalist program		
Program lead	3 (20.0)	5 (11.9)
Quality improvement lead	1 (6.7)	4 (9.5)
CME coordinator	1 (6.7)	2 (4.8)
Committee participant	6 (40.0)	17 (40.5)
Program scheduling	4 (26.7)	3 (7.1)
Other	6 (40.0)	19 (45.2)
Hospital type		
Rural hospital	0 (0.0)	0 (0.0)
Small community hospital	0 (0.0)	2 (4.8)
Medium community hospital	6 (40.0)	12 (28.6)
Large community hospital	4 (26.7)	15 (35.7)
Academic hospital	5 (33.3)	13 (31.0)
Average daily census of hospitalist program		
Less than 100 patients	0 (0.0)	5 (11.9)
101–120 patients	0 (0.0)	1 (2.4)
121–140 patients	3 (20.0)	6 (14.3)
141 or more patients	12 (80.0)	30 (71.4)
Number of FTE in the program		
Less than 10	1 (6.7)	6 (14.3)
11–15	1 (6.7)	7 (16.7)
15 or more	13 (86.7)	25 (59.5)
How many years since hospitalist program was implemented		
Less than 5	0 (0.0)	0 (0.0)
6–10	1 (6.7)	8 (19.0)
11–15	13 (86.7)	26 (61.9)
16 or more	1 (6.7)	2 (4.8)
Not known	0 (0.0)	6 (14.3)

CCFP = Certificate of the College of Family Physicians of Canada, CME = continuing medical education, FTE = full time equivalent

**Table 2:** Characteristics of participants in system redesign

**Characteristics**	***N* (%)**
Received pay for performance incentives	
Yes	1 (6.7)
No	14 (93.3)
Setting/level of system redesign/QI efforts	
Hospital	14 (93.3)
Private practice	1 (6.7)
Health authority	4 (26.7)
Provincial project	0 (0.0)
National project	0 (0.0)
Area of focus for QI project	
VTE prophylaxis	5 (20)
Preprinted order set development	5 (20)
Documentation and handovers	3 (12)
Urinary tract infections	2 (8)
Patient flow and care transitions	2 (8)
Comanagement of orthopedic patients	2 (8)
Handwashing	2 (8)
Other	4 (16)
Percentage of time spent on QI	
Less than 5%	13 (86.7)
5%–10%	1 (6.7)
10%–20%	1 (6.7)
20% or more	0 (0.0)
Received dedicated time for QI activities	
Yes	4 (26.7)
No	11 (73.3)
Received compensation for QI activities	
Yes	1 (6.7)
No	14 (93.3)

QI = quality improvement, VTE = venous thromboembolism

Table 3 ranks the facilitators for involvement in QI and system redesign efforts from the most (lowest score) to the least (highest score) important option according to survey respondents. Table 4 provides a similar analysis for barriers for participation. From the options provided to them, survey respondents identified dedicated time for QI, support from physician leadership, and financial incentives as the top three facilitators for involvement in QI. Conversely, lack of time and high clinical workload, lack of QI training and skills, and lack of access to data were identified as top barriers.

**Table 3:** Facilitators of engagement in quality improvement/system redesign

**Facilitator**	Score*
Dedicated time for QI or other nonclinical activities	3.4
Support from physician leadership (e.g., department chiefs)	4.8
Financial incentives	5.4
Formal training in QI concepts and methodologies	5.6
Data accessibility	6.5
Development of physician–organization “compact”—a shared understanding of mutual responsibilities and contributions	6.6
Being given the opportunity to take on leadership role in QI projects by management or physicians leaders	6.6
Support from physician colleagues	7.0
Performance measurement (e.g., physician scorecards)	7.4
Robust/easy to use electronic medical records	7.7
Support from hospital managers/administration	8.0
Data transparency (e.g., public reporting)	8.5
Skilled allied health-care providers	9.8
Academic promotion	11.6
Other	13.8

QI = quality improvement

^*^Lowest score denotes higher importance (1 = most important, 15 = least important)

**Table 4:** Barriers to participation in system redesign

**Barrier**	Score*
Lack of time and/or high clinical workload	1.4
Lack of quality improvement skills or training	3.6
Lack of access to external and internal performance data	3.9
Lack of support from hospital administration	5.1
Allied health-care staff turnover, staff inexperience, or lack	5.4
Lack of or difficult to use electronic medical reports	5.7
Cost	6.3
Desire for autonomy and individualized patient care	6.4
Lack of support from physicians colleagues of physicians leadership	6.4
Other	8.5

^*^Lower score denotes higher importance (1 = most important and 10 = least important)

### Qualitative findings

In addition to the rank-order questions, the free text responses from our survey participants provided a rich source of additional information. For example, respondents highlighted an adversarial relationship between hospitalists and hospital administrators as an important barrier to effective participation in QI work. They generally expressed a negative view of the hospital administration's motives in advocating for QI, and an overall negative environment where they felt hospitalists were not being recognized adequately for their contributions to the health system. Another theme that emerged was that many frontline physicians were simply not aware of the various QI projects underway in their institutions. They underscored the need for more effective efforts to build “awareness” of QI priorities and initiatives. Finally, an unexpected theme that emerged was the level of work experience and “seniority” of the hospitalists in relation to their colleagues, with some expressing concerns about leading projects when other more experienced members of the team would be required to change their practice patterns. This may highlight unique cultural issues among the hospitalists in our survey sample that can present a barrier to QI involvement by more junior members of the hospitalist programs. Other themes reinforced the results from the rank-order questions. Table 5 summarizes the results of our thematic analysis, and provides select quotes to illustrate observed themes.

**Table 5:** Thematic analysis of open-ended responses

**Theme**	**Number of mentions by survey respondents**	**Quotes**
Workload/lack of time	29	“Clinical work is totally consuming of available time.”
		“There is usually no time left at the end of a work day for any activities not pertaining strictly to patient care due to high patient care volume.”
		“Our hospitalist program is a very busy program and there is not a lot of time set aside for quality improvement strategies.”
		“The work load is heavy, and patient complexity is high, making hard to find time and energy to do these things.”
		“Too busy at work with teaching commitments and constant over census work load and shortages of staff leave no time to get involved in any projects.”
Relationship with administration	15	“Sometimes what really needs improvement is an aspect of the system over which we have no control as hospitalists, e.g., other dysfunctional departments and recruitment of particular specialists.”
		“Hospitals with large infrastructures are often slow to make changes/improvements due to delays in administration and larger groups of people to liaise with.”
		“We are viewed with disdain by hospital administration—even when there have been incredible improvements in flow (reduction in beds days for the short stay unit and the orthopedic patients), these are not appreciated in any manner, and in fact, are cut back!!”
		“It does not appear to be a priority for medical administration, any gains made have not be acknowledged by HA.”
		“End point for admin is cost saving and not high-quality care.”
		“Low morale due to lack of contract with no additional funding means nobody has any enthusiasm to take on projects that administration does not support.”
Financial compensation	12	“All efforts and time spent are not compensated and take time away from paid clinical work.”
		“Mostly just that time spent on QI is not remunerated so time must be made during (remunerated) patient care time.”
		“I don't think I would be financially compensated for these initiatives, so I'm more tempted to work where I'm getting reimbursed.”
QI training	7	“My lack of QI training/skills is an important barrier, as it makes such activities even more time-consuming.”
		“I have not had previous training in this area and would not know how to access data.”
Awareness	7	“Lack of awareness of what quality improvement initiatives are available.”
		“Need to be invited to participate in these initiatives.”
		“Hospital admin/physician leaders don't communicate about where needs are, and how they would like to see hospitalist input/involvement.”
Resources/data	6	“Lack of basic resources in terms of physical space allocated for hospitalists to meet and work out of, to plan and organize/evaluate effective QI measures.”
		“Difficult to evaluate opportunities for improvement because of difficulty in getting data.”
Experience/seniority	4	“I work in multiple settings outside of my hospitalist work and do not have a great deal of experience compared to my colleagues.”
		“As a more junior member, I also feel a bit intimidated with these projects and potentially pointing out flaws or mistakes made by both myself or colleagues.”
		“My experience with QI is very novel...could gain further learning and support among my colleagues.”
Competing priorities	2	“Many differing ideas among our group.”
Physician autonomy	2	“The desire for autonomy and individualized patient care is an important barrier, as guidelines are useful to a certain extent, but we are frequently faced with cases where such guidelines do not really apply, or the situation is quite complex and we need to use our judgment to individualize care.”
		“I'm sick of protocols and preprinted orders. I want to practice medicine, not check boxes.”
Leadership	1	“Hospitalists are in a good position to identify areas of patient care that need improvement. At the present time, these initiatives are spearheaded by nursing and others.”
Evidence of efficacy	1	“Lack of proven efficacy of many quality improvement initiatives.”
Physician engagement	1	“Lack of colleague participation.”

QI = quality improvement, HA = Health Authority

### Quantitative findings

To understand if there are any factors that may predict an individual hospitalist's participation in QI, we examined the relationship between QI participation and individual, programmatic, and institutional characteristics. In this analysis, participation in QI (question 16 of the questionnaire) was the dependent variable, and all other variables were treated as independent. Tables 6 and 7 summarize the results of this analysis. We conducted a logistic regression to control for interactions between predictors and their impact on participation in QI. In this analysis, the only significant variables were QI training and weeks worked as a hospitalist (*p* < 0.05). This suggests that the other factors entered into the model (gender, certification and training, years of experience as a physician and as a hospitalist, work status, and workload as measured by the number of patients seen daily) are not significantly contributing to QI participation.

**Table 6:** Engagement in quality improvement—univariate analysis

**Independent variable**	**χ^2^ test**	**λ**
Years practiced as hospitalist	8.5**	0
Work status	4.868*	0
Annual weeks worked as hospitalist	9.36**	0
Number of patients seen daily	4.798*	0
Formal training in QI	5.602**	0.067

QI = quality improvement

^*^p < 0.1,

^**^p < 0.05

**Table 7:** Determinants of quality improvement involvement—multivariate analysis

	**B**	**SE**	**Wald**	***df***	**Sig.**	**Exp (B)**
Years worked as physician	0.01	0.81	0.00	1.00	0.99	NS
Sex	−0.16	0.88	0.03	1.00	0.86	NS
Years worked as hospitalist	−1.51	0.84	3.23	1.00	0.07	NS
Credentials	−0.58	0.63	0.83	1.00	0.36	NS
Work status	16.32	10877.62	0.00	1.00	1.00	NS
Average daily patients seen	−0.56	0.51	1.21	1.00	0.27	NS
QI training	1.93	0.96	4.03	1.00	0.04	6.89
Weeks worked as hospitalist annually	−1.90	0.96	3.91	1.00	0.05	0.15
Constant	10.38	4.39	5.58	1.00	0.02	32298.64

QI = quality improvement, B = regression coefficient, SE = standard error, Wald = Wald statistics, *df* = degrees of freedom, Sig. = significance level (*p* value), Exp (B) = odds ratio, NS = Not Significant

## Discussion

QI and patient safety is considered an important cornerstone of hospital medicine.[25] Despite this, our understanding about individual, programmatic, and institutional characteristics that may facilitate or impair a hospitalist's involvement is limited. Knowing if there are certain attributes that facilitate QI participation by hospitalists is important on a number of levels. Locally, hospital medicine program leaders could select for individuals who are more likely to participate in QI when recruiting new physicians. Similarly, they could redesign their programs to specifically promote QI through structural changes (such as adjusting clinical workloads or changing compensation mechanisms). Armed with this knowledge, institutional leaders can take concrete actions on reducing real and perceived barriers to engagement, and provide support to individuals who are keen in taking part in QI. Finally, for policy makers and professional leaders who aim to promote QI more broadly, this knowledge could allow for development of specific programs (such as medical training curricula, changes to credentialing standards, or financial support for QI training).

Our quantitative analysis suggests that a number of attributes may be important in promoting hospitalist involvement in system change projects. Individuals who work full time appear to be more likely to engage in QI than those who work on a part-time basis. In addition, formal training in QI methodology was found to be associated with higher levels of participation. Previous studies have shown that individuals who received formal training in QI concepts express higher knowledge of QI concepts and more engaging attitudes.[26,27] Other factors (such as gender, age, credentials, size of the program, and type of hospital) do not appear to have a significant impact.

Our qualitative analysis largely supports the aforementioned findings, and sheds light on additional barriers and facilitators to physician engagement. Respondents identified lack of QI skills and training as the second most important barrier for participation in these efforts. This was further highlighted in the open-text answers, and they corroborated the findings of the logistic regression. Other top barriers to participation included high clinical workload and lack of time, as well as lack of access to performance data. On the contrary, having dedicated time for nonclinical activities, being supported by the physician leadership of the group, and financial incentives were deemed necessary facilitators of participation in system redesign efforts. Responses to the open-ended questions further reinforced these themes [Table 5].

Survey respondents also identified tensions between physicians and hospital administrators, lack of awareness of administration's priorities, and power differentials between senior and junior hospitalist team members as additional barriers. Differences in priorities and mindsets between hospital administrators and physicians have been identified as an important contributor to physicians' lack of engagement.[4,28] Other surveys of physicians in BC have also identified the relationship between physicians and health system managers as an area where improvements are needed.[29] Our respondents identified lack of awareness of institutional priorities as a challenge. The importance of communication as a necessary ingredient in engaging physicians has been previously described in the literature.[4] It appears that communication about priorities and organizational improvement needs is particularly important for hospitalists. Finally, an unexpected theme that has emerged in our research is the power differential between the more junior members of hospitalist groups and the more experienced ones. It is unclear if this hierarchical power dynamic is unique to our study population, or if this is a feature of the hospitalist model more broadly.

Our study has a number of notable limitations. First, the response rate to our survey (17.7%) is significantly lower than surveys of physicians reported in the literature.[30] A number of approaches have been suggested to improve response rates from physicians,[31] such as endorsement of surveys by professional societies, simple questionnaire design, and reminders. Although we used these strategies, the response rate might have been improved by incorporating monetary incentives or additional reminders. Our response rate may have significantly limited the power of our quantitative analysis. Despite this, there is still some evidence to suggest that factors such as formal training are sufficiently salient to merit further research. On the contrary, the qualitative component of our survey yielded a rich array of opinions from survey respondents. Future research should include other modalities (such as semi-structured interviews) to corroborate the findings from the survey responses.

Second, our survey was conducted on a very specific segment of the physician workforce in the province of BC, and as a result may not be generalizable to physicians working in other health-care settings (such as community clinics), those who do not engage in hospital medicine, and those in other geographic locations. However, other surveys in BC show some similar themes around barriers to engagement and suggest that hospitalist opinions may not be drastically different from the broader physician community in the province.

Third, similar to other survey studies, our research was not exempt from selection and sampling biases. For example, we identified an additional 93 individuals who engaged in hospitalist work in the province but were not on the Doctors of BC mailing list. In spite of this, we did obtain wide representation from different areas in the province, as well as hospitals of various sizes.

## Conclusion and Future Directions

Our findings are unique in a number of ways. To the best of our knowledge, our study was the first attempt to understand the various individual or organizational attributes that could facilitate involvement by hospitalists in QI activities. Although our statistical insights may be limited by a small sample size, our study suggests a number of potential characteristics that facilitate participation in QI and provides a starting point for future research. It highlights the factors that are perceived by hospitalists as important enablers of participation on change efforts, and points to the importance of formal training as a potential key facilitator for this group of physicians. Future research should focus on understanding the role of formal QI training in improving clinical outcomes for patients cared by hospitalists, and the various QI competencies that need to be included in effective educational programs.
