# Supplementary material for: Determinants of Hospital-based Physician Participation in Quality Improvement: A Survey of Hospitalists in British Columbia, Canada
Source: Glob J Qual Saf Healthc. 2020 Feb 6;3(1):6–13. doi: 10.4103/JQSH.JQSH_17_19 (PMC10335784; doi:10.4103/JQSH.JQSH_17_19)
Supplement: Supplementary file 1 [file i2589-9449-3-1-6_s01.doc]

**BC Hospitalist Quality Improvement Survey**

**1) Consent**

#### We intend for your participation in this project to be pleasant and stress-free. Your participation is entirely voluntary and you may refuse to participate or withdraw from the study at any time. By saying yes to this question, you consent to participating in this survey study

🔾 Yes
 🔾 No

**2) How long have you been participating in providing hospitalist care?**

 🔾 Less than 1 year
 🔾 1-2 years
 🔾 3-5 years
 🔾 6-10 years
 🔾 11 or more years

**3) Which one these options best describes your credentials?**

 🔾 CCFP with Additional Training (eg. Extra year in emergency medicine/anesthesia, or additional self-directed third year - minimum 3 months of additional rotations)
 🔾 CCFP - IMG (International Medical Graduate)
 🔾 FRCP - General Internal Medicine
 🔾 FRCP - Specialty Fellowship of Internal Medicine (e. cardiology etc)
 🔾 Other (please specify)

If you selected other, please specify ______________________________________________________________________

**4) Which of these options best describes your involvement with hospitalist work? (A “Dedicated Hospitalist” is defined as someone whose main clinical work is hospital medicine; “Full time” is defined as working at least 1650 hours annually as a hospitalist; “Locum Hospitalist” is someone whose main clinical work is something other than hospital medicine (eg. Primary care, maternity care, emergency medicine) but occasionally participates in hospitalist care).**

 🔾 Dedicated Hospitalist - Full Time
 🔾 Dedicated Hospitalist - Part Time
 🔾 Partial / Intermittent / Locum Hospitalist

**5) In a typical year, how many weeks do you spend in clinical activities (regardless of the setting such as hospital, office etc) ?**

 🔾 Less than 12 weeks
 🔾 13-24 weeks
 🔾 25-36 weeks
 🔾 37-48 weeks
 🔾 49 or more weeks

**6) In a typical year, how many weeks do you spend working in a hospitalist program?**

 🔾 Less than 12 weeks
 🔾 13-24 weeks
 🔾 25-36 weeks
 🔾 37-48 weeks
 🔾 49 or more weeks

**7) What is an average number of patients that you are responsible for on a daily basis (i.e. your individual census)**

 🔾 Less than 12 patients
 🔾 13-16 patients
 🔾 17-20 patients
 🔾 20 or more patients

**8) what is your role in your hospitalist program? (Select All That Apply)**

 ❑ Program Lead
 ❑ Quality Improvement Lead
 ❑ CME Coordinator
 ❑ Committee Participant
 ❑ Program Scheduling
 ❑ Other (please specify)

If you selected other, please specify ______________________________________________________________________

**9) Have you ever been trained formally in Quality Measurement or Improvement Principles?**

 🔾 Yes
 🔾 No

**10) What type of hospital is your hospitalist program located at? (If you work in more than one hospital, refer to where you spend the majority of your hospitalist work)**

 🔾 Rural Hospital
 🔾 Small Community Hospital
 🔾 Medium Size Community Hospital
 🔾 Large Community Hospital
 🔾 Academic Hospital

**11) Which health authorities do you work in? (select all that apply)**

 ❑ Fraser Health
 ❑ Vancouver Coastal
 ❑ Vancouver Island
 ❑ Northern Health
 ❑ Interior Health
 ❑ Provincial Health Services Authority

**12) What is an average daily census of your hospitalist program?**

 🔾 Less than 100
 🔾 101-120
 🔾 121-140
 🔾 141 or more
 🔾 I do not know

**13) What is the number of Full-Time Equivalent (FTE) hospitalists in your program?**

 🔾 Less than 10 FTE
 🔾 11-15 FTE
 🔾 15 FTE or more
 🔾 I do not know

**14) How many years ago was your hospitalist program established?**

 🔾 Less than 5 years
 🔾 6-10 years
 🔾 11-15 years
 🔾 16-20 years
 🔾 21 or more years
 🔾 I do not know

**15) Do you receive pay for performance (P4P) or bonuses based on clinical quality outcomes from your hospitalist program (eg. Incentives for better length of stay, timely discharge summaries etc)?**

 🔾 Yes
 🔾 No

**16) Some physicians have engaged in efforts to REDESIGN office or hospital systems to better manage patients' clinical care. Examples include developing systems to ensure that all abnormal tests are followed-up on or that all patients receive beta-blockers following heart attacks. In the past two years, have you been involved in any such efforts on local, regional, provincial or national levels?**

 🔾 Yes
 🔾 No

**17) Was this effort to redesign systems and procedures at the hospital or in your medical practice outside of the hospital? (Select all that apply)**

 ❑ Hospital
 ❑ Private Practice
 ❑ Health Authority
 ❑ Provinical Project
 ❑ National Project

**18) Please tell us the specific area and goal of your effort and how the goal was to be achieved.**

____________________________________________________________________________________________________________________________________________________________________________________________________________________________

**19) If you have been involved in quality improvement (QI) activities, what percentage of your time is spent on improvement efforts?**

 🔾 Less than 5%
 🔾 5-10 %
 🔾 10-20 %
 🔾 20 % or more

**20) Do you have dedicated time for QI activities?(i.e are you paid for the time you spend on quality improvement projects)?**

 🔾 Yes
 🔾 No

**21) If Yes, who provides the compensation you receive?**

 ❑ Hospital
 ❑ Health Authority
 ❑ Our own hospitalist group's internal arrangement
 ❑ I'm note sure

**22) How effective do you think each of the following would be in improving the quality of care you provide to your patients?**

|  | Not At All Effective | Not Very Effective | Somewhat Effective | Very Effective |
| --- | --- | --- | --- | --- |
| More use of computer technology such as electronic medical records and physician order entry | ❑ | ❑ | ❑ | ❑ |
| Better treatment guidelines or protocols for common conditions or procedures | ❑ | ❑ | ❑ | ❑ |
| Having more time to spend with your patients | ❑ | ❑ | ❑ | ❑ |
| Having better access to the best specialized physicians and services | ❑ | ❑ | ❑ | ❑ |
| Improved teamwork or communication among physicians and /or other medical care professionals | ❑ | ❑ | ❑ | ❑ |
| Having more access to resources such as more nursing staff, discharge planners and other allied healthcare | ❑ | ❑ | ❑ | ❑ |
| Better patient access to preventive care and health education | ❑ | ❑ | ❑ | ❑ |
| Having more access to resources such as more hospital beds, OR time, endoscopy or imaging equipment | ❑ | ❑ | ❑ | ❑ |
| Having dedicated/paid time to allocate to improvement work | ❑ | ❑ | ❑ | ❑ |
| Better communication between hospitalists and primary care physicians | ❑ | ❑ | ❑ | ❑ |

**23) Please rank in order of importance (1= most important and 10= least important) the reasons that act as BARRIERS for you to participate in formal quality improvement initiatives?**

| Lack of time and/or high clinical workload | ____________________ |
| --- | --- |
| Desire for autonomy and individualized patient care | ____________________ |
| Lack of quality improvement skills or training | ____________________ |
| Lack of access to external and internal performance data | ____________________ |
| Lack of or difficult to use Electronic Medical Reports (EMR) | ____________________ |
| Allied healthcare staff turnover, staff inexperience or lack of adequate numbers of non-physician providers | ____________________ |
| Cost | ____________________ |
| Lack of support from physicians colleagues of physicians leadership | ____________________ |
| Lack of support from hospital administration | ____________________ |
| Other (important barriers not listed here - Please see next question to elaborate) | ____________________ |

**24) Are there any other barriers that you have encountered that are not listed in the previous question?**

____________________________________________________________________________________________________________________________________________________________________________________________________________________________

**25) For your top 3 answers in Question 23, can you elaborate how these are barriers for you?**

____________________________________________________________________________________________________________________________________________________________________________________________________________________________

**26) For your top 3 answers in Question 23, can you identify solutions or strategies to mitigate these barriers? What can be done to remove these barriers from your list?**

____________________________________________________________________________________________________________________________________________________________________________________________________________________________

**27) Please rank in order of importance (1= most important and 15= least important) the reasons that act as FACILITATORS for you to participate in quality improvement?**

| Support from physician leadership (eg. Department Chiefs etc) | ____________________ |
| --- | --- |
| Being given the opportunity to take on leadership role in QI projects by management or physicians leaders | ____________________ |
| Development of physician -organization “compact” – a shared understanding of mutual responsibilities and contributions | ____________________ |
| Financial incentives | ____________________ |
| Performance measurement (eg. Physician scorecards) | ____________________ |
| Data transparency (eg. Public reporting) | ____________________ |
| Academic promotion | ____________________ |
| Dedicated time for QI or other non-clinical activities | ____________________ |
| Formal Training in QI concepts and methodologies | ____________________ |
| Data accessibility | ____________________ |
| Robust/easy to use Electronic Medical Records | ____________________ |
| Skilled allied health care providers | ____________________ |
| Support from physician colleagues | ____________________ |
| Support from hospital managers/administration | ____________________ |
| Other (important Facilitators not listed here - Please see next question to elaborate) | ____________________ |

**28) Are there any other facilitators that you have encountered that are not listed in the previous question?**

____________________________________________________________________________________________________________________________________________________________________________________________________________________________

**29) For your top 3 answers in Question 27, can you elaborate how these are facilitators for you?**

____________________________________________________________________________________________________________________________________________________________________________________________________________________________

**30) For your top 3 answers in Question 27, can you identify how these can be embedded in routine practice? How can these facilitators be used to make participation in QI activities a natural part of working as a hospitalist?**

____________________________________________________________________________________________________________________________________________________________________________________________________________________________

**31) Age**

 🔾 Less than 30
 🔾 30-40
 🔾 40-50
 🔾 50-60
 🔾 60-70
 🔾 Over 70

**32) Gender**

 ❑ Female
 ❑ Male

**33) How many years have you been a practicing medical doctor?**

 ❑ Less than a year
 ❑ 1-2 years
 ❑ 3-5 years
 ❑ 6-10 years
 ❑ 11-15 years
 ❑ 16 or more years

Thank you very much for participating in this study.
